# First report of *Aliarcobacter cryaerophilus* in ready-to-cook chicken meat samples from super shops in Bangladesh

**DOI:** 10.5455/javar.2023.j659

**Published:** 2023-03-31

**Authors:** Md. Muket Mahmud, Ajran Kabir, Md. Zawad Hossain, Sanjida Jamal Mim, Israt Jahan Yeva, Minara Khatun, Mohammad Saidur Rahman, Madan Mohan Dey, K. H. M. Nazmul Hussain Nazir

**Affiliations:** 1 Department of Microbiology and Hygiene, Bangladesh Agricultural University, Mymensingh, Bangladesh; 2 Department of Agricultural Economics, Bangladesh Agricultural University, Mymensingh, Bangladesh; 3 Department of Agricultural Sciences, Texas State University, San Marcos, TX, USA; † These two authors contributed equally.

**Keywords:** Aliarcobacter cryaerophilus, 16SrRNA, PCR, phylogenetic analysis, poultry meat, super shops

## Abstract

**Objective:**

This study aimed to isolate *Aliarcobacter cryaerophilus* in ready-to-cook poultry meat in Bangladesh.

**Materials and Methods:**

Thirty drumstick samples were collected from super shops in Dhaka city (*n* = 10), Mymensingh city (*n* = 10), and Patuakhali town (*n* = 10). After sample processing, they were cultured in Blood agar media with *Campylobacter* base using a microfilter (0.42 nm). Suspected colonies were subjected to DNA extraction and PCR assay targeting *16SrRNA* genes. Then, sequencing was performed for confirmation.

**Results:**

Of 30 samples, 3 (10%) were positive for *A. cryaerophilus*. Phylogenetic analysis shows that our isolate has strong similarities with one of the isolates from China.

**Conclusion:**

The presence of this organism in ready-to-cook poultry meat is a significant concern for consumers as it bears zoonotic importance.

## Introduction

*Aliarcobacter cryaerophilus* (previously *Arcobacter cryaerophilus*) is a foodborne and zoonotic pathogen increasing globally. *Aliarcobacter* (formerly *Arcobacter*) is a genus of the family *Campylobacteraceae* [1,2]. They can be distinguished from *Campylobacter *sp*.* by their ability to grow at 15°C. This genus has grown its importance in recent years as its members are considered emergent enteropathogens. The *Aliarcobacter* genus comprises nine Gram-negative species, which include* A. cryaerophilus, A. butzleri*, *A. cibarius*, *A. faecis*, *A. skirrowii, A. lanthieri*, *A. thereius*, *A. vitoriensis*, and *A. trophiarum* [1,3,4]. Among these*, A. cryaerophilus, A. butzleri*, *A. skirrowii *are considered to be zoonotic and foodborne pathogens that could cause illness in humans. *Aliarcobacter *species are typically found in poultry, beef, pig, seafood, and aquatic habitats [4–6]*.* Some sources of contamination include animal and human waste, fertilizer runoff, sewage backups, and animal defecation [7]. And *A.* *cryaerophilus *has pathogenic effects on humans and animals, which may cause gastroenteritis, bacteremia, sepsis, mastitis, diarrhea, abortion, and reproductive disorders [8].

Till now, they have been found in food of animal origin, particularly in poultry, carcasses and offal, milk, and mussels, as well as in water bodies, sewage, and feces of many animal species. The presence of these organisms can be a significant threat to public health and a primary concern for food safety issues. However, minimal data are available on the prevalence of *Aliarcobacters *spp*.* in ready-to-cook chicken meat. In addition, there is no standard procedure for the culture and isolation of *Aliarcobacters* spp. Therefore, this study aimed to isolate and characterize *A. cryaerophilus* based on the *16SrRNA *gene from poultry meat (drumstick) samples.

## Materials and Methods

### Ethical approval

The protocols of this study were approved by the Animal Welfare and Experimentation Ethics Committee, Bangladesh Agricultural University, Mymensingh [approval number AWEEC/BAU/2020(36)].

### Sample collection

Thirty frozen chicken meat (Drumstick) samples were collected randomly from selective supermarkets in Dhaka city (*n* = 10), Mymensingh city (*n* = 10), and Patuakhali town (*n *= 10) in Bangladesh. Aseptic methods were used to collect the meat samples, which were then transferred adequately to sterile containers. Cool chain was maintained to transport the samples to the Bacteriology Laboratory at the Department of Microbiology and Hygiene, BAU, Mymensingh.

### Culture and staining

*Aliarcobacter* spp. was isolated using the filtration method [9]. Samples were prepared by separating 1 gm of each meat sample suspended in 900 μl of PBS. Blood agar bases, including Campylobacter supplements, were used for isolation, and a 0.45 um filter paper was placed on each agar plate. 100 μl of the suspension was spread onto the surface filters using the drop method, and the drops were permitted to stand for 30 min at room temperature. After 30 min, the filter was removed and incubated at 37°C for 48 h in the anaerobic jar. Selected colonies were subjected to Gram stain.

### Molecular detection of Aliarcobacter spp. and nucleotide sequencing

Following the method described by Shahid et al. [10], DNA was extracted from the pure colonies. The genus of *Aliarcobacter* was confirmed using *16s rRNA *gene primers by PCR, as described in the previous study [9].

The PCR reaction used the set of primers 16S9F (Anti-sense: 5’- GAG TTT GAT CCT GGC TC-3”) and 16S1540R (Sense: 5’- AAG GAG GTG ATC CAG CC-3”) to amplify a fragment of 1,530 base pairs. The composition of the mix for each reaction with 25 μl of the final volume was: 1 μl (20 pmol) of each primer, 2 ul of (100 ng/ul) of genomic DNA, and 2× of Go *Taq* Green Master Mix (Promega, USA). The amplification was performed at 47°C annealing temperature for 30 sec. After electrophoresis on 1.2% agarose gel, ethidium bromide staining was performed and subjected to gel documentation under UV light.

### Phylogenetic analysis 

One PCR product was analyzed by forward and reversed sequencing with a Sanger sequencing technology. Chromas version 2.5 and MEGA X software were used for sequence assembly. The sequence was compared with the GenBank database using the BLASTn [11]. Seven strains of *A. cryoaerophilus *and one* A. skirrowii* strain were selected based on percent identity for phylogenetic analysis. Multiple sequence alignment was performed in CLUSTALW [12].

Furthermore, the phylogenetic tree was constructed using the Neighbor-Joining method. The *p*-distance method was used for evolutionary distance, and the bootstrap value was 1,000 [13]. The nucleotide sequence generated in this study has been deposited in the GenBank.

## Results

### Occurrence of A. cryaerophilus

**Figure 1.**
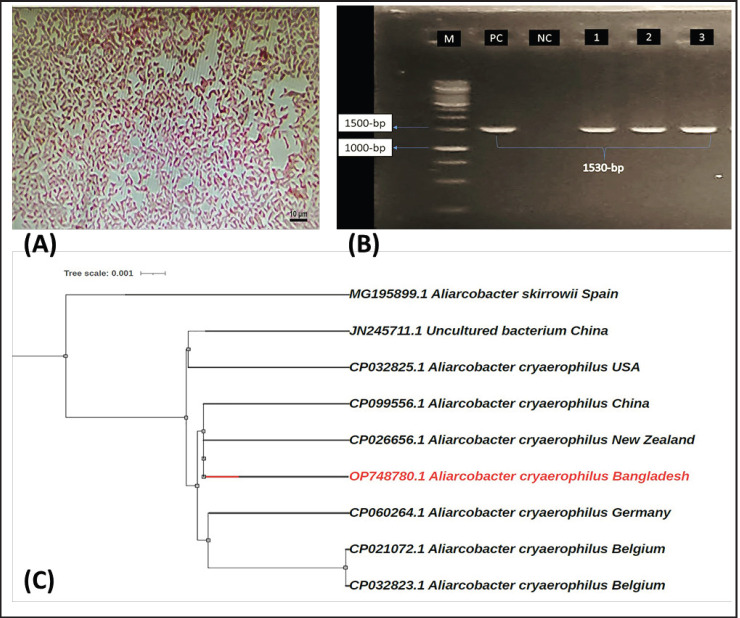
Identification and phylogenetic analysis of *A. cryaerophilus. *(A) Gram-stain of *A. cryaerophilus *showing Gram-negative curve shape at 100× magnification, (B) PCR-identification of *Aliarcobacter *sp*.*; Lane M: 100-bp DNA ladder, PC: Positive control, NC: Negative control, and Lane 1-3: *16SrRNA* gene identification, representing PCR amplification of *Aliarcobacter *spp., (C) *16SrRNA* gene-based phylogenetic analysis of the isolate using the Neighbor-Joining method with 1,000-bootstrap replicates through MEGA-X software.

Out of 30 chicken meat samples, 3 (10%) were culturally positive (Table 1). They formed grey, flat, and irregularly spreading colonies on Blood agar with Campylobacter agar base. Gram-negative curves were observed under the microscope (Fig. 1). Culturally positive samples were further subjected to PCR targeting *16SrRNA* genes, and 1,530-bp amplicons were found in gel electrophoresis (Fig. 1).

### PCR and nucleotide sequencing

The PCR amplicons were visualized in 1.2% gel under UV light, and 1,530-bp band sizes were found (Fig. 1). After sequencing and completion of forward and reverse sequence alignment, a 1,449-bp length sequence was found for further study. Nucleotide BLAST was performed for sequence validation, and our query sequence produced significant alignment with available *A. cryaerophilus * (Table 2) with substantial similarities.

### Phylogenetic analysis

A total of eight sequences were downloaded from NCBI, including in-group and out-group, to construct a phylogenetic tree for our isolate (Table 2). The neighbor-joining tree was constructed, and our sequence shares the same clade with two other isolates of *A. cryoaerophilus* belonging to China and New Zealand (Fig. 1).

### Data availability

*16SrRNA* gene fragment sequence of *A. cryaerophilus* KHMN_BAU1 isolate is available in NCBI with the accession number OP748780.

**Table 1.** Occurrence of *Aliarcobacter cryaerophilus. *

No	Group	No. of samples	No of *16s rRNA* gene positive samples	Prevalence
**1**	Dhaka	10	2	20%
**2**	Mymensingh	10	0	0
**3**	Patuakhali	10	1	10%
**4**	Overall	30	3	10%

**Table 2.** Validation table and list of sequence obtained from Gene Bank (NCBI).

Accession	Organism	Strain	Country	Query coverage	E Value	% Identity
In group						
CP026656.1	*Aliarcobacter_cryaerophilus*	M83MA	New Zealand	99%	0	99.72%
CP099556.1	*Aliarcobacter_cryaerophilus*	ICDEAC48	China	99%	0	99.72%
CP032825.1	*Aliarcobacter_cryaerophilus*	D2610	USA	99%	0	99.65%
CP060264.1	*Aliarcobacter_cryaerophilus*	16CS0369-1-AR-4	Germany	99%	0	99.65%
JN245711.1	*Uncultured bacterium*	MY-95	China	99%	0	99.58%
CP021072.1	*Aliarcobacter_cryaerophilus*	LMG9904	Belgium	99%	0	99.10%
CP032823.1	*Aliarcobacter_cryaerophilus*	ATCC 43158	Belgium	99%	0	99.10%
Out group						
MG195899.1	*Aliarcobacter_skirrowii*	LMG 6021	Spain	99%	0	98.68%

## Discussion

*Aliarcobacter* (previously *Arcobacter*) prevalence is noticeable in food, especially in poultry worldwide. This concern led us to conduct this study to ensure the presence of this organism in ready-to-cook chicken meat available in super shops in Bangladesh. This study has found a 10% occurrence of *Aliarcobacter *spp*.* in chicken meat purchased from super shops of selected regions in Bangladesh. Further, sequencing has confirmed the *A. cryaerophilus*. This is the first report of *A. cryaerophilus* in chicken meat(drumstick) in Bangladesh.

This incidence rate supports the previous findings in Iran, where it was observed at 8.66% [14]. Besides, the prevalence of *Aliarcobacter* spp. in chicken meat was reported in India (58%) [15], Japan (60%) [16], Korea (45.8%) [17], and Malaysia (39.2%) [18]. The possible reason for this low prevalence might be the ready-to-cook chicken meat in supermarkets, and multiple washing steps reduce the contamination possibilities of these meats [19].

Approximately 90% of the broiler carcasses are contaminated with arcobacters, particularly with *A. butzleri* and *A. cryaerophilus* [20,21]. Several studies have been conducted worldwide, and most of them have reported the presence of this organism on the skin and the slaughter processing unit of poultry [22,23]. It is assumed that the contamination of *A. cryaerophilus *in ready-to-eat drumsticks might have occurred from the food processing unit or the carcass of those chickens, a significant public health concern and one health issue*. *

Culture, PCR, and sequencing have been used in this study to isolate and detect this organism. *16s rRNA *gene-specific primer of *Campylobacter *sp*.* has been used as they share the same family [1,7]. It is challenging to differentiate *Campylobacter* and *Aliarcobacter* using cultural and genus-specific PCR assays. Thus, sequencing was performed for the confirmation of *A. cryareophilus*.

Phylogenetic analysis revealed that our isolate is closely related to isolates of China (CP099556.1). It is relatable that the organism can be derived directly from China as lots of food items and poultry shed utensils are imported every year [24].

Ready-to-cook poultry meat is considered the safest product based on its processing. However, *A. cryaerophilus-*contaminated meat can infect humans during handling and cooking, which may lead to self-limiting diarrhea [25]. Again, undercooked meat can lead to foodborne illness. Thus, a comprehensive study is required, especially from the slaughterhouse, including their surrounding environmental samples, to elucidate the transmission pattern of these bacteria. Moreover, the antimicrobial resistance profile of these bacteria should be studied to develop the prevention and control of this zoonotic genus.

## Conclusion

*Aliarcobacter cryaerophilus *has been detected in ready-to-cook poultry meat (Drumstick) for the first time in Bangladesh. It is suggested to adopt hygienic measures when handling and cooking such food items.

## List of abbreviations

gm, grams; μl, microliter; PBS, phosphate buffere solution; um, micrometer; minutes, minutes; DNA, deoxyribonucleic acids; ng, nanograms; UV, ultraviolet; MEGA, Molecular evolutionary genetics analysis; BLAST, The Basic Local Alignment *Search* Tool; NCBI, National Center for Biotechnology Information.

## References

[1] Pérez-Cataluña A, Salas-Massó N, Diéguez AL, Balboa S, Lema A, Romalde JL (2018). Revisiting the taxonomy of the genus *Arcobacter*: getting order from the chaos. Front Microbiol.

[2] Niedermeyer JA, Miller WG, Yee E, Harris A, Emanuel RE, Jass T (2020). Search for *Campylobacter* spp. reveals high prevalence and pronounced genetic diversity of *Arcobacter butzleri* in flood water samples associated with Hurricane Florence in North Carolina, USA. Appl Environ Microbiol.

[3] Khan I, Becker A, Cloutier M, Plötz M, Lapen D, Wilkes G (2021). Loop‐mediated isothermal amplification: development, validation and application of simple and rapid assays for quantitative detection of species of *Arcobacteraceae* family‐and species‐specific *Aliarcobacter faecis* and *Aliarcobacter lanthieri*. J Appl Microbiol.

[4] Munson E, Carroll KC. (2019). An update on the novel genera and species and revised taxonomic status of bacterial organisms described in 2016 and 2017. J Clin Microbiol.

[5] Chieffi D, Fanelli F, Fusco V. (2020). *Arcobacter butzleri*: Up‐to‐date taxonomy, ecology, and pathogenicity of an emerging pathogen. Compr Rev Food Sci Food Saf.

[6] Çelik C, Pınar O, Sipahi N. (2022). The prevalence of *Aliarcobacter* species in the fecal microbiota of farm animals and potential effective agents for their treatment: a review of the past decade. Microorganisms.

[7] Chuan J, Belov A, Cloutier M, Li X, Khan IU, Chen W. (2022). Comparative genomics analysis and virulence-related factors in novel *Aliarcobacter faecis* and *Aliarcobacter lanthieri* species identified as potential opportunistic pathogens. BMC Genom.

[8] Isidro J, Ferreira S, Pinto M, Domingues F, Oleastro M, Gomes JP (2020). Virulence and antibiotic resistance plasticity of *Arcobacter butzleri*: insights on the genomic diversity of an emerging human pathogen. Infect Genet Evol.

[9] Neogi SB, Islam M, Islam S, Akhter A, Sikder M, Hasan M (2020). Risk of multi-drug resistant *Campylobacter *spp. and residual antimicrobials at poultry farms and live bird markets in Bangladesh. BMC Infect Dis.

[10] Shahid AH, Nazir KHMNH, El Zowalaty ME, Kabir A, Sarker SA, Siddique MP (2021). Molecular detection of vancomycin and methicillin resistance in Staphylococcus aureus isolated from food processing environments. One Health.

[11] Hossain MJ, Raut S, Singh RP, Mishra P, Hossain MS, Dey AR (2022). Molecular detection of Babesia and Theileria from crossbred cattle in Sirajganj and Rangpur districts of Bangladesh. Vet Med Sci.

[12] Thompson JD, Gibson TJ, Higgins DG. (2003). Multiple sequence alignment using ClustalW and ClustalX. Curr Protoc Bioinformatics.

[13] Jahan MS, Rahman MM, Ahmed R, Kabir A, Oli S, Hassan J (2021). Molecular characterization of duck plague virus for determination of TCID 50. Res Agric Livest Fish.

[14] Khodamoradi S, Abiri R. (2020). The incidence and antimicrobial resistance of *Arcobacter* species in animal and poultry meat samples at slaughterhouses in Iran. Iran J Microbiol.

[15] Ramees TP, Rathore RS, Bagalkot PS, Ravi Kumar GVPPS MH, Anoopraj R, Kumar A (2014). Real-time PCR detection of *Arcobacter butzleri* and *Arcobacter cryaerophilus* in chicken meat samples. J Pure Appl Microbiol.

[16] Ohnishi T, Hara‐Kudo Y. (2021). Presence and quantification of pathogenic *Arcobacter* and *Campylobacter* species in retail meats available in Japan. Lett Appl Microbiol.

[17] Kim NH, Park SM, Kim HW, Cho TJ, Kim SH, Choi C (2019). Prevalence of pathogenic *Arcobacter* species in South Korea: comparison of two protocols for isolating the bacteria from foods and examination of nine putative virulence genes. Food Microbiol.

[18] Amare L, Saleha A, Zunita Z, Jalila A, Hassan L. (2011). Prevalence of *Arcobacter* spp. on chicken meat at retail markets and in farm chickens in Selangor, Malaysia. Food Contr.

[19] Shumaker ET, Kirchner M, Cates SC, Shelley L, Goulter R, Goodson L (2022). Observational study of the impact of a food safety intervention on consumer poultry washing. J Food Prot.

[20] On SL, Miller WG, Biggs PJ, Cornelius AJ, Vandamme P. (2020). A critical rebuttal of the proposed division of the genus *Arcobacter* into six genera using comparative genomic, phylogenetic, and phenotypic criteria. Syst Appl Microbiol.

[21] Kunert-Filho HC, Furian TQ, Sesterhenn R, Chitolina GZ, Willsmann DE, Borges KA (2022). Bacterial community identification in poultry carcasses using high-throughput next generation sequencing. Int J Food Microbiol.

[22] Martins I, Mateus C, Domingues F, Oleastro M, Ferreira S. (2023). Putative role of an ABC efflux system in *Aliarcobacter butzleri* resistance and virulence. Antibiotics.

[23] Kumar H, Bhardwaj K, Kaur T, Nepovimova E, Kuča K, Kumar V (2020). Detection of bacterial pathogens and antibiotic residues in chicken meat: a review. Foods.

[24] (2022). Bangladesh Total Imports from China. https://www.ceicdata.com/en/indicator/bangladesh/total-imports-from-china.

[25] Uljanovas D, Gölz G, Brückner V, Grineviciene A, Tamuleviciene E, Alter T (2021). Prevalence, antimicrobial susceptibility and virulence gene profiles of *Arcobacter* species isolated from human stool samples, foods of animal origin, ready-to-eat salad mixes and environmental water. Gut Path.

